# Frontal QRS-T angle in patients with Graves’ disease: association with thyroid function and cardiovascular parameters

**DOI:** 10.3389/fendo.2026.1979248

**Published:** 2026-10-22

**Authors:** Maksymilian Kłosowicz, Magdalena Urbańczuk, Aleksandra Burbelka, Agnieszka Gala-Błądzińska, Krzysztof Balawender

**Affiliations:** 1 Department of Normal and Clinical Anatomy, Faculty of Medicine, Medical College, University of Rzeszów, Rzeszów, Poland; 2 Clinical Department of Cardiology with the Acute Coronary Syndromes Subdivision, St. Queen Jadwiga Clinical Hospital in Rzeszow, Rzeszow, Poland; 3 Department of Internal Medicine, Nephrology and Endocrinology, St. Queen Jadwiga Clinical Hospital in Rzeszow, Rzeszow, Poland; 4 Department of Nephrology and Endocrinology, Faculty of Medicine, Medical College, University of Rzeszów, Rzeszów, Poland; 5 Department of Nephrology and Dialysis Unit, Fryderyk Chopin University Clinical Hospital in Rzeszów, Rzeszów, Poland

**Keywords:** cardiac electrophysiogy, electrocardiagram (ECG), frontal QRS-T angle, Grave’s disease (GD), overt hyperthyroidism, ventricular depolarization, ventricular repolarization

## Abstract

**Introduction:**

Thyroid hormone excess may alter ventricular electrophysiology, affecting both depolarization and repolarization. The frontal QRS-T (fQRS-T) angle reflects the directional relationship between these processes and may provide information on ventricular electrical heterogeneity. However, data on the fQRS-T angle in overt hyperthyroidism (OHT) remain limited. We evaluated the fQRS-T angle in patients with Graves’ disease presenting with OHT compared with euthyroid controls.

**Materials and methods:**

This retrospective single-center study included 55 patients with Graves’ disease and biochemically confirmed OHT and 42 euthyroid controls. Frontal QRS and T axes were calculated from ECG measurements in leads I and aVF. Between-group comparisons, multivariable regression adjusted for age, sex, body mass index, and heart rate, sensitivity analyses, ROC analysis, and correlations with thyroid function parameters were performed. Results: The median fQRS-T angle was greater in patients with OHT than in controls (21.89° [12.37–44.09] vs. 17.36° [9.02–27.29], p = 0.006), with a rank-biserial correlation of 0.328 (95% CI 0.106–0.535). An fQRS-T angle >45° occurred in 23.6% of patients with OHT and in none of the controls (p < 0.001), whereas the QRS and T axes did not differ significantly between groups. After adjustment for age, sex, body mass index, and heart rate, OHT remained associated with a greater fQRS-T angle (β = 9.00°, 95% CI 1.77–16.22; p = 0.015), with consistent findings in sensitivity analyses. Discriminatory performance was modest (AUC 0.664, 95% CI 0.552–0.768). No significant associations were observed between fQRS-T angle and FT3, FT4, TSH, or heart rate.

**Discussion:**

Patients with Graves’ disease and OHT exhibited greater discordance between ventricular depolarization and repolarization despite similar individual QRS and T axes. This association persisted after adjustment for heart rate and other potential confounders, suggesting that fQRS-T widening may reflect electrophysiological alterations associated with OHT rather than tachycardia alone.

**Conclusion:**

Overt hyperthyroidism due to Graves’ disease is associated with a wider fQRS-T angle independently of age, sex, body mass index, and heart rate. Larger prospective, multicenter studies with longitudinal ECG assessment are needed to determine the reversibility and prognostic significance of these alterations.

## 1 Introduction

Overt hyperthyroidism (OHT) is a state of thyroid hormone excess characterized biochemically by suppressed serum thyroid-stimulating hormone (TSH) concentrations accompanied by elevated levels of free thyroxine (FT4) and/or free triiodothyronine (FT3) above the respective reference ranges (1). Disturbances in thyroid hormone (TH) homeostasis exert systemic effects, with the cardiovascular system being particularly sensitive to changes in thyroid function (2). Excess thyroid hormones induce a broad spectrum of cardiovascular alterations, including changes in hemodynamics and cardiac electrophysiology, and are associated with an increased risk of heart failure and cardiac arrhythmias (3).

Electrical abnormalities associated with OHT can be assessed using a standard 12-lead electrocardiogram (ECG). Thyroid hormone excess may affect both ventricular depolarization and repolarization, thereby altering the electrical properties of the myocardium (4, 5). Our previous studies have shown that OHT is associated with alterations in conventional ECG parameters and indices reflecting ventricular repolarization heterogeneity (6). Although a recent study in patients with Graves’ disease demonstrated an association between an increased fQRS-T angle and hyperthyroid heart disease, the relationship between the fQRS-T angle and individual thyroid function parameters and heart rate has not been extensively characterized (7).

The QRS-T angle is an electrocardiographic parameter reflecting the angular difference between the directions of ventricular depolarization and repolarization. An increased QRS-T angle indicates greater discordance between these electrical processes and may therefore reflect impaired ventricular electrical homogeneity. Accordingly, this parameter may provide additional information on electrophysiological alterations that are not fully captured by conventional ECG indices (8).

The primary aim of the present study was to compare the frontal QRS-T angle between patients with overt hyperthyroidism due to Graves’ disease and euthyroid controls. Secondary objectives included assessment of the frontal QRS and T axes, evaluation of associations between thyroid function parameters and the fQRS-T angle, and determination of whether the association between OHT and fQRS-T angle persisted after adjustment for age, sex, BMI, and heart rate.

## 2 Methods

### 2.1 Data collection

Data collection was performed at a tertiary referral center located in southeastern Poland. The study protocol complied with the principles of the Declaration of Helsinki and received approval from the local institutional ethics committee (approval no. KB/23/D/2025).

Demographic, anthropometric, and laboratory data were obtained from the hospital electronic medical records and complemented, when necessary, with information available in paper-based documentation. The collected variables were transferred to a study-specific Microsoft Excel database, where they were coded and structured for subsequent statistical analysis. To ensure confidentiality, all records were anonymized and assigned unique identification numbers.

Available 12-lead electrocardiograms were retrieved from the patients’ medical documentation. When more than one ECG tracing was available for a given patient, the recording obtained closest in time to the blood sample used for assessment of thyroid function was selected for analysis.

### 2.2 Study population

#### 2.2.1 Study group

The study included patients aged ≥18 years who were hospitalized between June 1, 2022, and June 30, 2025, and had a documented history of hyperthyroidism due to Graves’ disease. Overt hyperthyroidism was defined as a suppressed serum TSH concentration accompanied by FT4 and/or FT3 concentrations above the respective laboratory reference ranges. Based on these initial eligibility criteria, 953 potentially eligible patients were identified. The predefined exclusion criteria, detailed in **Table 1**, were subsequently applied to this cohort. Following the selection process, 55 patients met all eligibility requirements and were included in the final analysis.

**Table 1:** Exclusion criteria and the number of patients excluded at each stage of the study population selection process.

Exclusion criteria	Number of patients
History of heart disease^1^	157
Absence of thyroid hormone measurements in medical records	100
Thyroid hormone levels not indicative of current overt hyperthyroidism	102
Electrolyte imbalance^2^	64
Comorbid endocrine or systemic disorders	83
Use of medications affecting myocardial function or cardiac electrophysiology	267
Age <18 years or >85 years	33
Lack of ECG	13
Incomplete medical history	80

1 History of heart disease, including coronary artery disease, previous myocardial infarction, heart failure, cardiomyopathy, clinically significant valvular heart disease, congenital heart disease, clinically significant cardiac arrhythmias, and cardiac conduction disorders.

2 Electrolyte imbalance, defined as serum sodium (reference range: 135–145 mmol/L), potassium (3.5–5.1 mmol/L), or chloride (98–107 mmol/L), calcium (8.6–10 mg/dL) concentrations outside the respective laboratory reference ranges.

Throughout the manuscript, patients with Graves’ disease presenting with overt hyperthyroidism are referred to as the overt hyperthyroidism (OHT) group.

#### 2.2.2 Control group

The control group consisted of individuals who presented to the hospital for reasons unrelated to thyroid and cardiac diseases. Participants were selected to ensure the absence of significant systemic diseases that could potentially affect electrocardiographic parameters. Euthyroidism was defined as serum TSH, FT3, and FT4 concentrations within the respective laboratory reference ranges. Individuals with a history of thyroid disease were excluded. The same predefined exclusion criteria regarding cardiovascular disease, electrolyte disturbances, relevant comorbidities, and medications potentially affecting cardiac electrophysiology were applied to both study groups. Inclusion criteria of control group are presented in **Table 2**.

**Table 2:** Inclusion criteria of patients included to control group.

Inclusion criteria
Lack of history of heart diseases^1^
Lack of history of thyroid diseases or prior thyroid-specific treatment^3^
Euthyroidism in laboratory tests^4^
Electrolyte imbalance^2^
Significant renal, hepatic, inflammatory, or other systemic diseases
Age between 18 years and 85 years
Lack of medications affecting myocardial function or cardiac electrophysiology^5^
ECG tracing quality: adequate for analysis
Complete medical history

1 History of heart disease, including coronary artery disease, previous myocardial infarction, heart failure, cardiomyopathy, clinically significant valvular heart disease, congenital heart disease, clinically significant cardiac arrhythmias, and cardiac conduction disorders.

2 Electrolyte imbalance, defined as serum sodium (reference range: 135–145 mmol/L), potassium (3.5–5.1 mmol/L), or chloride (98–107 mmol/L), calcium (8.6–10 mg/dL) concentrations outside the respective laboratory reference ranges.

3 Lack of history of thyroid diseases, cardiovascular diseases, diabetes mellitus, chronic kidney disease, chronic liver disease, significant pulmonary diseases, systemic inflammatory or autoimmune diseases, active malignancy, and other clinically significant systemic disorders that could affect cardiac electrophysiology.

4 Euthyroidism was defined as serum TSH, FT3, and FT4 concentrations within the laboratory reference ranges: TSH [0,27 – 4,2] mIU/L, FT3 [2,0-4,4] pg/mL, and FT4 [0,93-1,71] ng/dL.

5 Medications potentially affecting myocardial function or cardiac electrophysiology, including antiarrhythmic agents, β-blockers, non-dihydropyridine calcium channel blockers, cardiac glycosides, sympathomimetic or sympatholytic agents, and other drugs known to affect cardiac conduction, automaticity, contractility, or ventricular repolarization (including QT-prolonging medications).

### 2.3 ECG analysis

Standard 12-lead ECG recordings of 10 seconds were obtained at a paper speed of 25 mm/s and a amplitude calibration of 10 mm/m. Digital ECG measurements were subsequently performed with ImageJ software (National Institutes of Health, Bethesda, MD, USA). Before each analysis, the measurement scale was standardized according to the ECG grid, using five large horizontal divisions as a 1-second (1000-ms) reference and a vertical distance of 10 mm as the equivalent of 1 mV.

All ECG-derived variables were linked to the respective patient identification codes and recorded in a structured study-specific Excel database for subsequent statistical analysis.

The frontal QRS-T angle (fQRS-T angle) was determined manually from standard 12-lead electrocardiograms. For each tracing, the amplitudes of the QRS complex and T wave were measured in leads I and aVF using ImageJ software (National Institutes of Health, Bethesda, MD, USA), with the isoelectric line used as the reference level. The net QRS amplitude was calculated as the algebraic sum of positive and negative QRS deflections, whereas T-wave amplitude was measured from the isoelectric line to its peak, with positive and negative deflections assigned the corresponding sign. Measurements obtained from three consecutive cardiac cycles were averaged.

The method used to determine the QRS complex and T-wave amplitudes is illustrated in **Figure 1**.

**Figure 1:**
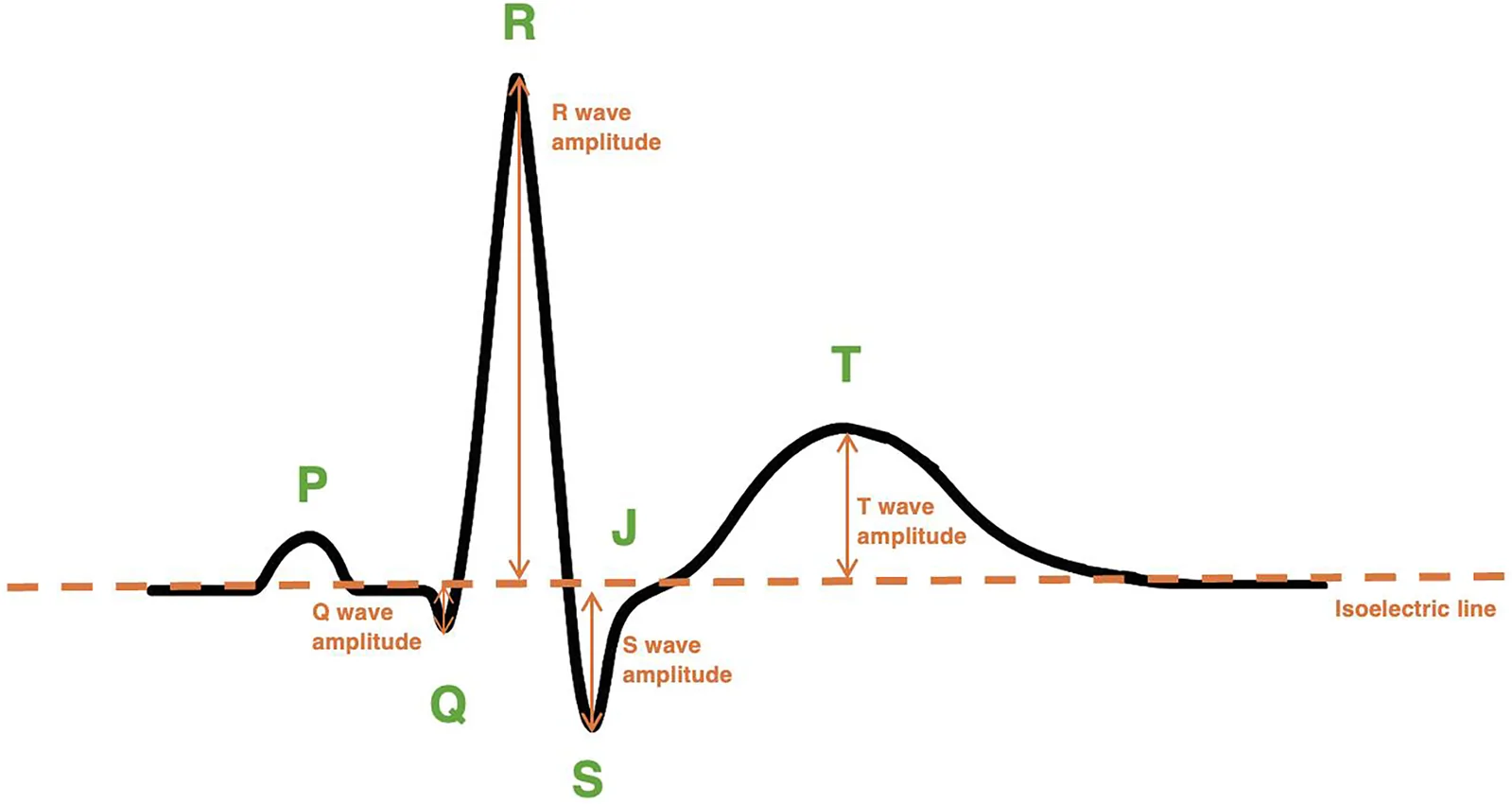
Schematic representation of electrocardiographic wave amplitudes used for frontal QRS–T angle calculation. Q-, R-, S-, and T-wave amplitudes were measured relative to the isoelectric line. Q and S waves were assigned negative values, whereas the R wave was assigned a positive value. T-wave amplitude was recorded according to its direction relative to the isoelectric line. Measurements were obtained in leads I and aVF and subsequently used to calculate the frontal QRS and T axes.

The frontal electrical axes of the QRS complex and T wave were subsequently calculated from the respective amplitudes in leads I and aVF using the two-argument arctangent function (ATAN2), with angular values expressed in degrees. The absolute difference between the calculated QRS and T axes was then determined. When this difference exceeded 180°, the fQRS-T angle was calculated as 360° minus the absolute angular difference, yielding values ranging from 0° to 180°. Schematic representation of the frontal QRS-T angle is presented on **Figure 2**.

**Figure 2:**
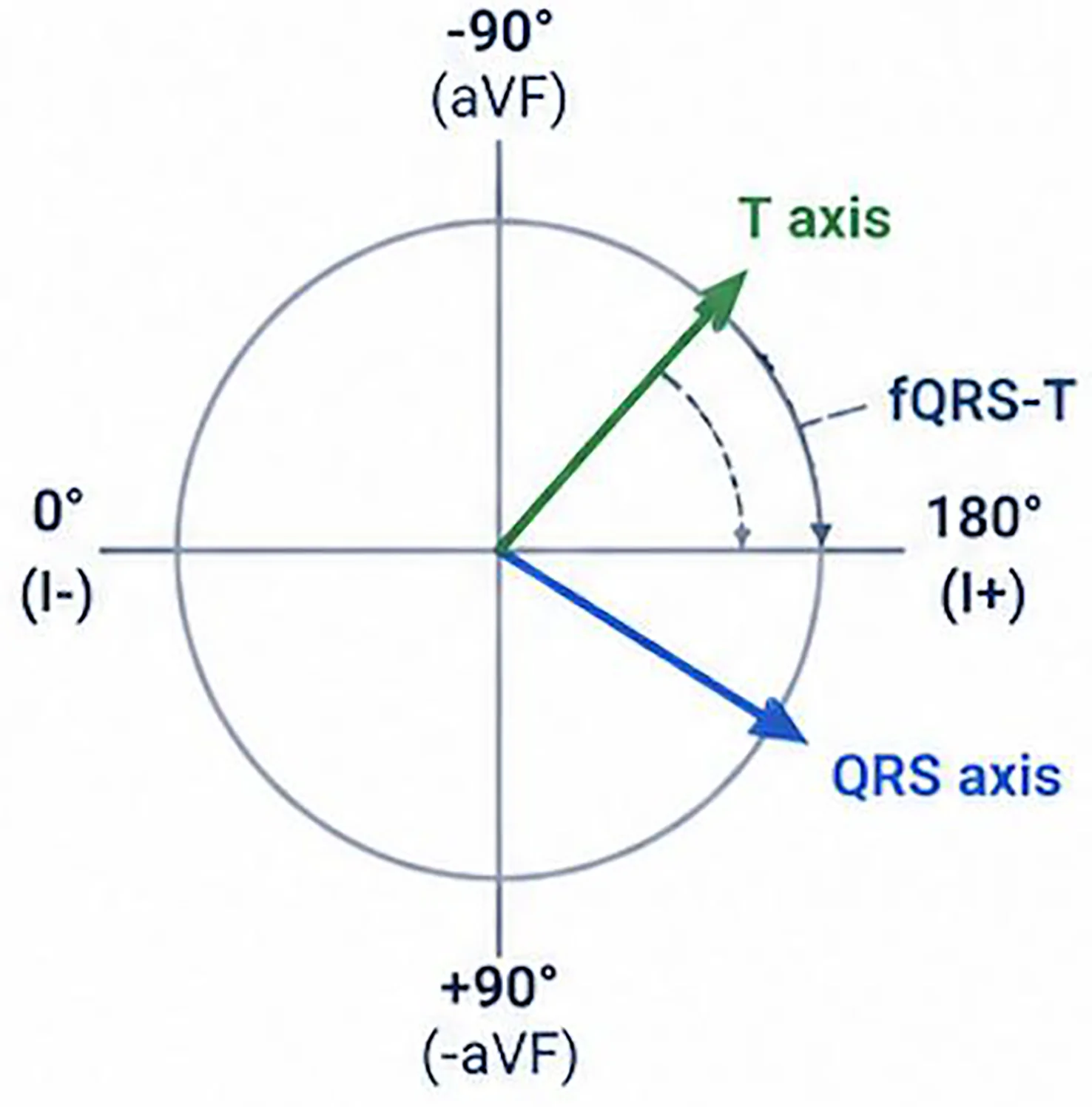
Schematic representation of the frontal QRS-T angle. The diagram illustrates the frontal plane coordinate system, with lead I defining the horizontal axis and aVF the vertical axis, and shows the spatial orientation of the QRS and T-wave electrical axes and the resulting angular separation between them. The QRS and T-wave axes are calculated from the respective net amplitudes in leads I and aVF using the two-argument arctangent function (ATAN2), with angular values expressed in degrees. The figure also shows the 0°, +90°, −90°, and 180° reference directions and the smaller angular difference representing the fQRS-T angle.

### 2.4 Statistical analysis

#### 2.4.1 Variables and estimands

The primary exposure was overt hyperthyroidism (OHT), with euthyroid status as the reference category. The primary outcome was the frontal QRS-T (fQRS-T) angle, analyzed as a continuous variable. Secondary electrocardiographic outcomes included the frontal QRS axis and T axis, while fQRS-T thresholds of >45° and >90° were assessed in exploratory categorical analyses. These thresholds were selected based on previously published classifications of the frontal QRS-T angle, in which values below 45°, 45–90°, and above 90° have been described as normal, borderline, and abnormal, respectively.

Age, sex, body mass index, and heart rate were included as potential confounders in adjusted models. The principal adjusted estimand was the difference in fQRS-T angle associated with OHT after accounting for these covariates. FT3, FT4, TSH, and heart rate were additionally evaluated as exploratory correlates of the fQRS-T angle within the OHT group.

#### 2.4.2 Descriptive statistics and group comparisons

Continuous variables were assessed for normality using the Shapiro–Wilk test and visual inspection of their distributions. Normally distributed variables are presented as mean ± standard deviation (SD), whereas non-normally distributed variables are reported as median with interquartile range [IQR]. Categorical variables are presented as counts and percentages.

Between-group comparisons were performed using the independent-samples t-test or Mann–Whitney U test, as appropriate. Categorical variables were compared using the chi-square test or Fisher’s exact test when expected cell counts were small. All tests were two-sided, and a p-value <0.05 was considered statistically significant.

#### 2.4.3 Analysis of frontal QRS-T angle and electrical axes

The fQRS-T angle was analyzed as a continuous variable. Owing to its non-normal distribution, the primary comparison between OHT and euthyroid controls was performed using the Mann–Whitney U test. The magnitude of the between-group difference was quantified using rank-biserial correlation, with 95% confidence intervals estimated using non-parametric bootstrap resampling.

The proportions of participants with fQRS-T angles >45° and >90° were compared using Fisher’s exact test. Because the frontal QRS and T axes represent directional data, between-group differences in their mean directions were additionally evaluated using circular statistical methods with permutation-based inference.

#### 2.4.4 Multivariable and sensitivity analyses

To assess whether the observed difference in fQRS-T angle was independent of potential confounding factors, multivariable regression analysis was performed with fQRS-T angle as the dependent variable and study group, heart rate, age, sex, and BMI as independent variables.

Given the right-skewed distribution of fQRS-T angle values, sensitivity analyses were performed using logarithmically transformed fQRS-T values and a generalized linear model with a gamma distribution and log link. A study group and heart rate interaction term was additionally examined to assess whether the association between heart rate and fQRS-T differed between groups.

#### 2.4.5 ROC and correlation analyses

Receiver operating characteristic (ROC) analysis was performed to explore the discriminatory ability of the fQRS-T angle for distinguishing patients with OHT from euthyroid controls. The area under the ROC curve (AUC) was calculated with a 95% confidence interval. An exploratory threshold was identified by maximizing the Youden index, with corresponding sensitivity and specificity calculated.

Within the OHT group, associations of the fQRS-T angle with FT3, FT4, TSH, and heart rate were evaluated using Spearman’s rank correlation coefficients. The Benjamini–Hochberg procedure was used to control the false discovery rate in exploratory correlation analyses.

#### 2.4.6 Measurement reproducibility

Measurement reproducibility was assessed using a two-way mixed-effects intraclass correlation coefficient (ICC) for absolute agreement based on single measurements, with corresponding 95% confidence intervals. Intraobserver agreement was evaluated in a randomly selected subset of 60 participants, including 30 patients with OHT and 30 euthyroid controls, by repeating QRS-axis, T-axis, and fQRS-T angle measurements under identical conditions.

## 3 Results

### 3.1 Study population and baseline characteristic

A total of 97 participants were included in the final analysis, comprising 55 patients with Graves’ disease and 42 euthyroid controls. The groups were comparable with respect to age, sex distribution, and body mass index (all p > 0.05). As expected, patients with OHT exhibited biochemical features of thyroid hormone excess. Detailed demographic, clinical, and laboratory characteristics of the study population are presented in **Table 3**.

**Table 3:** Baseline demographic and clinical characteristics of the study population.

Variable	Overt hyperthyroidism (n=55)	Euthyroid controls (n=42)	p-value
Age, years	46.0 [35.5–59.0]	46.5 [38.5–57.8]	0.746
Female sex, n (%)	43 (78.2)	28 (66.7)	0.250
BMI, kg/m²	21.78 [20.52–24.30]	22.75 [21.28–23.68]	0.675
Heart rate, beats/min	102 [94–113.5]	74.4 [65.9–85.1]	p <0.001

Data are presented as median [interquartile range] or number (percentage), as appropriate. BMI, body mass index; TSH.

In addition to thyroid function parameters, selected laboratory variables potentially relevant to ventricular electrical activity were assessed, including indices of renal function, hematological parameters, serum electrolytes, and cardiac biomarkers. Glucose and lipid parameters were additionally available in a subset of patients with OHT. eGFR was automatically calculated by the hospital laboratory, and the corresponding results were retrieved from the hospital information system. Detailed biochemical and laboratory characteristics of both study groups, together with the corresponding between-group comparisons, are presented in **Table 4**.

**Table 4:** Biochemical and laboratory characteristics of patients with overt hyperthyroidism and euthyroid controls.

Variable	OHT, n	OHT, median [IQR]	Euthyroid controls, n	Euthyroid controls, median [IQR]	p-value
Thyroid function parameters					
FT3, pg/mL	55	8.52 [5.49–14.79]	42	2.95 [2.50–3.375]	<0.001
FT4, ng/dL	55	3.28 [2.44–4.73]	42	1.10 [1.00–1.40]	<0.001
TSH, µIU/mL	55	0.007 [0.007–0.011]	42	1.74 [1.48–2.41]	<0.001
Metabolic and lipid parameters					
Glucose, mg/dL	45	103 [92–112]	—	—	—
Total cholesterol, mg/dL	17	126 [115–171]	—	—	—
LDL cholesterol, mg/dL	17	76 [61–98]	—	—	—
HDL cholesterol, mg/dL	20	35.75 [31.75–45.00]	—	—	—
Non-HDL cholesterol, mg/dL	14	98.5 [81.5–106.0]	—	—	—
Triglycerides, mg/dL	17	98 [85–120]	—	—	—
Renal function					
Creatinine, mg/dL	53	0.65 [0.47–0.80]	42	0.925 [0.863–0.990]	<0.001
eGFR, mL/min/1.73 m²	55	112 [97–120]	42	109.5 [101.25–115.75]	0.753
Hematological parameters					
Hemoglobin, g/dL	53	12.9 [12.1–14.1]	42	13.7 [12.9–14.7]	0.009
Hematocrit, %	53	38.0 [35.1–40.8]	—	—	—
Electrolytes					
Sodium, mmol/L	48	139.5 [138.0–141.0]	42	140.5 [138.25–144.0]	0.140
Potassium, mmol/L	48	4.20 [4.00–4.40]	42	4.20 [3.80–4.70]	0.691
Chloride, mmol/L	48	106.0 [103.75–107.25]	42	101.0 [100.0–104.0]	<0.001
Calcium, mg/dL	22	9.40 [9.21–9.74]	42	9.45 [9.13–9.70]	0.887
Cardiac biomarkers					
NT-proBNP, pg/mL	40	120.9 [108.4–132.6]	38	110.6 [101.4–119.8]	0.003
hs-cTnT, pg/mL	15	13.1 [12.3–14.2]	10	7.6 [7.2–8.1]	<0.001

Data are presented as median [interquartile range]. Between-group comparisons were performed using the Mann–Whitney U test. Dashes indicate that corresponding data were not available for the control group. OHT, overt hyperthyroidism; FT3, free triiodothyronine; FT4, free thyroxine; TSH, thyroid-stimulating hormone; LDL, low-density lipoprotein; HDL, high-density lipoprotein; eGFR, estimated glomerular filtration rate; NT-proBNP, N-terminal pro-B-type natriuretic peptide; hs-cTnT, high-sensitivity cardiac troponin T.

As expected, FT3 and FT4 concentrations were significantly higher and TSH concentrations significantly lower in patients with OHT than in euthyroid controls (all p < 0.001). Serum creatinine and hemoglobin concentrations were lower in the OHT group (p < 0.001 and p = 0.009, respectively), whereas no significant between-group differences were observed in eGFR or serum sodium, potassium, and calcium concentrations. Serum chloride concentrations were higher in patients with OHT (p < 0.001). NT-proBNP and hs-cTnT concentrations were also higher in the OHT group than in euthyroid controls (p = 0.003 and p < 0.001, respectively); however, these biomarkers were available only in subsets of participants.

### 3.2 Frontal QRS-T angle and electrical axes

Patients with Graves’ disease demonstrated significantly greater frontal QRS-T angles than euthyroid controls. The median fQRS-T angle was 21.89° [12.37–44.09] in the OHT group compared with 17.36° [9.02–27.29] in the control group (p = 0.006, Mann–Whitney U test; **Figure 3**). The rank-biserial correlation was 0.328 (95% bootstrap CI 0.106–0.535), indicating a small-to-moderate between-group effect.

**Figure 3:**
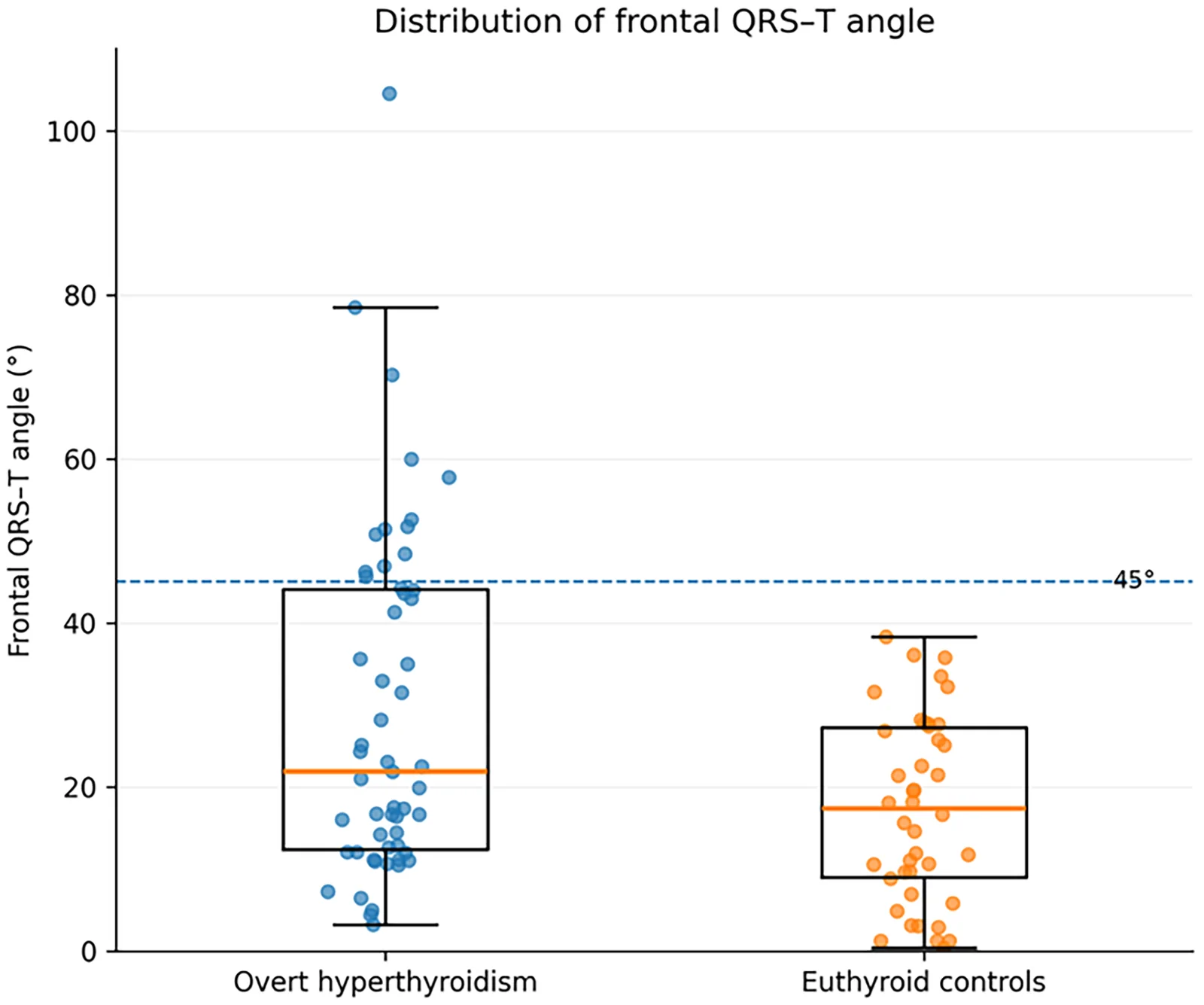
Distribution of frontal QRS-T angle in patients with overt hyperthyroidism and euthyroid controls. Individual data points are shown together with the median and interquartile range. The dashed horizontal line indicates a frontal QRS-T angle of 45°. Between-group differences were assessed using the Mann–Whitney U test.

In contrast, no significant between-group differences were observed for the frontal QRS and T axes considered separately. The median QRS axis was 35.5° [14.7–61.6] in patients with OHT and 39.0° [12.4–55.4] in controls (p = 0.774), whereas the corresponding T-axis values were 32.4° [21.9–55.9] and 32.6° [18.7–49.0], respectively (p = 0.675). Circular analyses similarly showed no significant differences in the mean direction of either the QRS or T axis between the groups (both p > 0.70).

A frontal QRS-T angle >45° was observed in 13 of 55 (23.6%) patients with OHT, whereas none of the 42 euthyroid controls exceeded this threshold (Fisher’s exact p < 0.001). An fQRS-T angle >90° was uncommon, occurring in only one patient with OHT and in none of the controls, with no significant between-group difference.

Receiver operating characteristic (ROC) analysis showed modest discriminatory ability of the frontal QRS-T angle for distinguishing patients with OHT from euthyroid controls, with an area under the curve (AUC) of 0.664 (95% CI 0.552–0.768). The exploratory threshold maximizing the Youden index was 41.3°, corresponding to a sensitivity of 32.7% and a specificity of 100%. Given the exploratory nature of this analysis and the absence of external validation, this threshold should not be interpreted as a clinically validated cut-off.

### 3.3 Multivariable analysis

Given the substantially higher heart rate observed in patients with OHT, multivariable analysis was performed to determine whether the between-group difference in frontal QRS-T angle was independent of heart rate and other potential confounders. After adjustment for heart rate, age, sex, and BMI, OHT remained significantly associated with a greater frontal QRS-T angle (adjusted β = 9.00°, 95% CI 1.77–16.22; p = 0.015). Heart rate itself was not independently associated with the frontal QRS-T angle in the adjusted model (β = 0.132° per beat/min; p = 0.170).

Given the right-skewed distribution of frontal QRS-T angle values, the robustness of this association was further evaluated using alternative model specifications. Following logarithmic transformation of the fQRS-T angle, the association with OHT remained significant (geometric mean ratio 1.68, 95% CI 1.10–2.54; p = 0.015). Consistent results were obtained using a generalized linear model with a gamma distribution and log link (ratio 1.58, 95% CI 1.14–2.18; p = 0.006). No significant interaction between OHT status and heart rate was observed (p for interaction = 0.479).

### 3.4 Associations with renal function and electrolyte parameters

Serum creatinine and chloride concentrations differed significantly between the OHT and euthyroid control groups, whereas eGFR and serum sodium, potassium, and calcium concentrations were comparable between the groups (**Table 4**). Within the OHT group, none of the evaluated renal function or electrolyte parameters, including creatinine, eGFR, sodium, potassium, chloride, and calcium, showed a significant correlation with the frontal QRS-T angle. None of these associations remained significant after correction for multiple comparisons (**Table 5**). These findings suggest that the observed wider fQRS-T angle in the OHT group was not readily attributable to variation in renal function or serum electrolyte concentrations.

**Table 5:** Correlations of renal function and serum electrolyte parameters with the frontal QRS-T angle in patients with overt hyperthyroidism.

Parameter	OHT, n	Spearman’s ρ	p-value	FDR-adjusted p
Creatinine, mg/dL	53	−0.057	0.683	0.819
eGFR, mL/min/1.73 m²	55	−0.098	0.475	0.819
Sodium, mmol/L	48	0.089	0.548	0.819
Potassium, mmol/L	48	0.101	0.494	0.819
Chloride, mmol/L	48	−0.013	0.929	0.929
Calcium, mg/dL	22	−0.115	0.610	0.819

Correlations were assessed using Spearman’s rank correlation. The Benjamini–Hochberg procedure was applied to control the false discovery rate (FDR) across correlation analyses. OHT, overt hyperthyroidism; eGFR, estimated glomerular filtration rate.

### 3.5 Associations with thyroid function and heart rate

Within the OHT group, the frontal QRS-T angle was not significantly correlated with biochemical indices of thyroid dysfunction. No significant associations were observed with FT3 (Spearman’s ρ = 0.129, p = 0.348), FT4 (ρ = 0.243, p = 0.074), or TSH (ρ= −0.169, p = 0.218). Similarly, the frontal QRS-T angle was not significantly correlated with heart rate (ρ = 0.136, p = 0.321). None of these associations remained significant after correction for multiple comparisons.

### 3.6 Measurement reproducibility

Intraobserver reproducibility of electrocardiographic measurements was assessed in a randomly selected subset of 60 participants (30 patients with OHT and 30 euthyroid controls) by repeating the measurements under identical conditions. Intraobserver reproducibility was excellent across all evaluated ECG measurements, with ICC values ranging from 0.94 to 0.97. Detailed results of the reproducibility analysis, including ICC values and their corresponding 95% confidence intervals, are presented in **Table 6**.

**Table 6:** Intraobserver reproducibility of frontal electrocardiographic axis measurements.

Parameter	ICC	95% CI	Interpretation
QRS axis	0.97	0.94–0.99	Excellent
T axis	0.96	0.92–0.98	Excellent
QRS-T angle	0.94	0.88–0.97	Excellent

## 4 Discussion

The principal finding of the present study is that patients with Graves’ disease exhibited a significantly wider frontal QRS-T angle than euthyroid controls. Importantly, this difference was observed despite the absence of significant between-group differences in the frontal QRS and T axes considered separately. This suggests that the observed widening of the fQRS-T angle may reflect an altered relationship between the directions of ventricular depolarization and repolarization rather than a uniform shift in either electrical axis alone.

The association remained significant after adjustment for age, sex, BMI, and heart rate, with OHT being associated with an approximately 9° greater fQRS-T angle. This finding is particularly relevant given the markedly higher heart rate observed in the OHT group. Heart rate itself was not independently associated with the fQRS-T angle in the adjusted model, and no significant interaction between OHT status and heart rate was identified. Moreover, sensitivity analyses accounting for the right-skewed distribution of fQRS-T values yielded consistent results. Collectively, these observations suggest that the wider fQRS-T angle observed in OHT cannot be explained solely by the accompanying increase in heart rate.

Another notable finding was the substantially higher prevalence of fQRS-T angles >45° among patients with OHT, whereas no control participant exceeded this threshold. However, markedly abnormal values >90° were rare, indicating that the electrical alterations observed in this cohort were generally modest rather than extreme. Consistent with this interpretation, ROC analysis demonstrated only modest discriminatory performance of the fQRS-T angle. Therefore, the present findings should not be interpreted as supporting its use as a diagnostic marker of hyperthyroidism, but rather as evidence of altered ventricular electrical characteristics associated with the hyperthyroid state.

Finally, no significant correlations were observed between the fQRS-T angle and FT3, FT4, or TSH concentrations within the OHT group. Although FT4 showed a weak positive trend, it did not reach statistical significance. The absence of a clear dose-response relationship with circulating thyroid hormone concentrations may indicate that the fQRS-T angle reflects a more complex electrophysiological response to thyroid hormone excess rather than the magnitude of biochemical abnormalities at a single time point. This interpretation should nevertheless remain cautious given the relatively limited sample size and the cross-sectional nature of the analysis.

These findings are broadly consistent with the observations reported by Wang et al., who investigated 291 patients with Graves’ disease and found that an fQRS-T angle ≥90° was significantly more prevalent in this population, whereas none of the 96 healthy controls exceeded this threshold. Moreover, during the 5-year follow-up, an fQRS-T angle ≥90° was associated with an approximately five-fold higher risk of first hospitalization for heart failure (7).

A widened QRS-T angle has been associated with adverse cardiovascular outcomes across a broad range of populations. Previous studies have demonstrated that an increased spatial QRS-T angle is associated with a higher risk of sudden cardiac death (SCD), ventricular arrhythmias, cardiovascular mortality, and all-cause mortality. Importantly, similar prognostic associations have also been reported for the frontal QRS-T angle. In a large Finnish population-based cohort of 10,957 middle-aged individuals, a frontal QRS-T angle >100° was associated with an approximately three-fold increased risk of SCD and a 1.8-fold higher risk of all-cause mortality. Moreover, analysis of 7,052 participants without baseline cardiovascular disease demonstrated an association between an abnormal frontal QRS-T angle and cardiovascular mortality, although this relationship appeared to vary according to sex. Collectively, these findings suggest that widening of the QRS-T angle may reflect increased electrical heterogeneity of the ventricles and may serve as a readily obtainable electrocardiographic marker associated with adverse cardiovascular outcomes (8, 9).

Alterations in the QRS-T angle have also been reported in several non-cardiovascular conditions. Increased fQRS-T values have been observed in patients with metabolic and endocrine disorders, including diabetes mellitus, subclinical hypothyroidism, and primary hyperparathyroidism, as well as in other systemic conditions such as chronic inflammatory diseases. These observations suggest that QRS-T angle widening may not be restricted to primary cardiac disorders but may also reflect myocardial electrical alterations secondary to systemic metabolic, hormonal, or inflammatory disturbances (10–14).

The pathophysiological mechanisms underlying QRS-T angle widening in hyperthyroidism remain incompletely understood. The QRS-T angle reflects the spatial or directional discordance between ventricular depolarization and repolarization, with greater angular differences indicating increased electrical heterogeneity of the ventricular myocardium. Thyroid hormones may contribute to altered ventricular electrophysiology through modulation of cardiac ion-channel expression, including voltage-gated potassium channels involved in repolarization (15, 16). In addition, enhanced adrenergic sensitivity and thyroid hormone-induced alterations in myocardial electrophysiology may modify regional repolarization sequences, thereby increasing the discrepancy between depolarization and repolarization vectors (17, 18).

Beyond its electrophysiological effects, thyroid hormone excess induces substantial hemodynamic and myocardial changes, including increased cardiac output, reduced systemic vascular resistance, and enhanced myocardial contractility. Persistent exposure to these alterations may promote adverse cardiac alterations and contribute to the development of clinically relevant cardiovascular complications in patients with hyperthyroidism (3).

To date, evidence regarding the frontal QRS-T angle in hyperthyroidism remains scarce. Wang et al. previously demonstrated an increased prevalence of markedly widened fQRS-T angles in patients with Graves’ disease and showed that an fQRS-T angle ≥90° was associated with adverse cardiovascular outcomes. The present study addresses a different, complementary question by evaluating whether active, biochemically confirmed overt hyperthyroidism is associated with alterations in the fQRS-T angle in a carefully selected population without established cardiac disease, electrolyte abnormalities, or medications affecting cardiac electrophysiology. Rather than focusing primarily on markedly abnormal fQRS-T values, we analyzed the angle as a continuous measure of ventricular electrical discordance. Notably, a significant between-group difference was detectable despite most patients with OHT having fQRS-T angles well below 90°, suggesting that alterations in depolarization–repolarization alignment may also be present at less pronounced degrees of fQRS-T widening. Furthermore, the association between OHT and a wider fQRS-T angle persisted after adjustment for age, sex, BMI, and heart rate and remained robust across alternative model specifications. Thus, our findings extend the observations of Wang et al. by indicating that modest alterations in the fQRS-T angle are detectable in overt hyperthyroidism even in the absence of established cardiac disease and cannot be explained solely by the accompanying increase in heart rate. The clinical and prognostic significance of these more subtle electrical alterations remains to be established.

From a clinical perspective, the fQRS-T angle may provide complementary information beyond conventional ECG parameters by integrating the spatial relationship between ventricular depolarization and repolarization into a single measure. In our study, neither the QRS axis nor the T-wave axis differed significantly between patients with OHT and controls when assessed separately, whereas the fQRS-T angle was significantly wider in the OHT group. This suggests that the fQRS-T angle may capture subtle abnormalities in the coordination between ventricular depolarization and repolarization that are not apparent from conventional assessment of individual ECG axes. Its use may therefore provide an additional dimension of electrical characterization in patients with overt hyperthyroidism, complementing standard ECG measures such as heart rate, QRS duration, and repolarization indices. In this context, the fQRS-T angle may represent a simple, readily obtainable marker of altered relationship between ventricular depolarization and repolarization that could be incorporated into routine ECG assessment without requiring additional testing or specialized equipment. Future studies should determine whether this additional electrical information can improve identification of patients at increased cardiovascular risk and provide clinically relevant information beyond conventional ECG and clinical parameters.

## 5 Conclusion and future directions

Patients with Graves’ disease presenting with overt hyperthyroidism exhibited a significantly wider frontal QRS-T angle than euthyroid controls, and this association remained significant after adjustment for age, sex, BMI, and heart rate. These findings suggest that overt hyperthyroidism may be associated with altered ventricular electrical properties reflected by greater discordance between depolarization and repolarization. However, the modest discriminatory performance of the fQRS-T angle and the absence of significant associations with thyroid hormone concentrations indicate that it should not be considered a diagnostic marker of OHT.

Future prospective, multicenter studies involving larger and more diverse populations are needed to confirm these findings and assess their generalizability. Longitudinal ECG evaluation before and after restoration of euthyroidism would help determine whether the observed electrical alterations are reversible. Further studies should also investigate whether fQRS-T angle widening is associated with subsequent arrhythmic or cardiovascular outcomes and whether it may provide incremental prognostic information in patients with overt hyperthyroidism.

## 6 Limitations

This study has several limitations. First, the relatively small sample size and single-center, retrospective design may limit the generalizability of the findings. The exact duration of overt hyperthyroidism prior to hospitalization was unknown, precluding assessment of the potential impact of cumulative thyroid hormone exposure on the fQRS-T angle. Although the analyses were adjusted for major potential confounders, including age, sex, BMI, and heart rate, residual confounding cannot be excluded. An additional limitation is that the cross-sectional design precluded assessment of the reversibility of the observed ECG alterations following restoration of euthyroidism, as well as their potential association with subsequent arrhythmic or cardiovascular outcomes.

Finally, the selection of the control group represents an additional limitation. Euthyroid controls were retrospectively identified among patients presenting to the emergency department who underwent both electrocardiography and thyroid function testing. Although strict eligibility criteria were applied to exclude individuals with known cardiovascular disease, relevant electrolyte abnormalities, or other conditions and medications expected to substantially affect cardiac electrophysiology, the emergency department setting itself may represent a potential source of selection bias. Acute illnesses, transient physiological disturbances, or other factors related to the reason for emergency department presentation may have influenced ECG parameters even in otherwise eligible individuals. Furthermore, the requirement for both ECG and thyroid function testing may have resulted in the inclusion of a selected subgroup that is not fully representative of the general euthyroid population. Therefore, the control group should not be regarded as a population-based reference sample for defining normal fQRS-T angle values. These considerations may limit the generalizability of the observed between-group differences, and validation in prospectively recruited healthy or community-based euthyroid controls is warranted.

## Data Availability

The raw data supporting the conclusions of this article will be made available by the authors, without undue reservation.
